# Evaluation of the Impact of a Regional Educational Advertising Campaign on Harm Perceptions of E-Cigarettes, Prevalence of E-Cigarette Use, and Quit Attempts Among Smokers

**DOI:** 10.1093/ntr/ntz236

**Published:** 2019-12-14

**Authors:** Harry Tattan-Birch, Sarah E Jackson, Charlotte Ide, Linda Bauld, Lion Shahab

**Affiliations:** 1 Department of Behavioural Science and Health, University College London, London, UK; 2 Cancer Research UK, London, UK; 3 Usher Institute, College of Medicine and Veterinary Medicine, University of Edinburgh, Edinburgh, UK

## Abstract

**Introduction:**

We evaluated how effective an advertising campaign that was piloted by Cancer Research UK in January/February 2018 was at promoting quit attempts by increasing awareness of the relative harms of e-cigarettes compared with smoking.

**Methods:**

Adults (≥16 years, *n* = 2217) living in Greater Manchester (campaign region), Yorkshire & Humber and the North East of England (control regions) completed cross-sectional surveys immediately before and after the campaign period. Surveys measured socio-demographics, perceptions and use of e-cigarettes, and motivation and attempts to quit smoking. We tested interactions between time (pre, post) and region (campaign, control).

**Results:**

36.7% (95% CI 33.0%–40.6%) of those in the intervention region recognized the campaign. In the general population, interactions were nonsignificant for all outcomes except for perception of e-cigarettes as effective cessation aids, with smaller increases from pre- to post-campaign in the campaign (49.9% to 54.0%) compared with the control region (40.5% to 55.0%; odds ratio [OR] = 0.66, 95% CI .45–0.98). Among smokers, motivation to quit increased in the intervention region (44.0% to 48.0%) but decreased in the control region (40.5% to 21.5%; OR = 2.97, 95% confidence interval [CI] 1.25–7.16), with no other significant differences between regions over time. Bayes factors confirmed that nonsignificant results were inconclusive.

**Conclusions:**

Compared with the control region, the campaign was associated with an increase in smokers’ motivation to quit but a smaller increase in adults’ perception of e-cigarettes as an effective cessation aid. There was insufficient evidence to determine whether the campaign affected other outcomes.

**Implications:**

Past extended mass media tobacco control campaigns have been shown to change public attitudes towards smoking, improve motivation to quit smoking, and reduce smoking prevalence. Much less is known about shorter, targeted campaigns. Here we show that using mass media to communicate accurate information about the relative harms of e-cigarettes compared with smoking may be an effective strategy in increasing smokers’ motivation to quit. Moreover, even when only run for a month, such campaigns can reach a large proportion of the targeted population. Further research is needed to evaluate effects on quit attempts and success.

## Introduction

Tobacco smoking is associated with substantial risk of premature morbidity and mortality^1^ and is the leading preventable cause of cancer, causing around 7 in 10 lung cancer cases in the United Kingdom.^2^ This excess risk is primarily driven by the inhalation of toxins contained within the smoke^3^ and could be mitigated by quitting or switching to nicotine products that do not contain tobacco or require combustion.^4^ Electronic cigarettes (e-cigarettes) are handheld electronic devices that allow the user to inhale nicotine in a vapor, without tobacco or combustion,^5^ thereby reducing nicotine cravings and withdrawal symptoms.^6^ However, public perceptions of e-cigarettes are inaccurate and have worsened over recent years even among smokers,^6,7^ which may discourage use. Increasing awareness of the relative harms of e-cigarettes and tobacco smoking is important to enable smokers to make informed decisions about product use. In this study, we evaluate the effectiveness of an advertising campaign designed to address misperceptions around e-cigarette harms relative to tobacco smoking.

Following a period of rapid growth in the prevalence of e-cigarette use (“vaping”) from 2011 to 2014, e-cigarettes are now used by around 2.6 million people in England (20% of smokers) and are the most common aid to smoking cessation, used in over one-third of quit attempts.^8^ Evidence from several randomized controlled trials indicates that using e-cigarettes in a quit attempt increases the chances of successful cessation.^9,10^ On a population level, the rise in use of e-cigarettes in England and the United States has been associated with increases in the overall success rate of quit attempts in the population,^11,12^ contributing to continued declines in smoking prevalence.^13^ While the prevalence of e-cigarette use in England has remained fairly stable since late 2013, use among long-term ex-smokers has continued to rise^14^ and some data suggest that there are now more ex-smokers than smokers using e-cigarettes.^15^

Although the long-term effects of using e-cigarettes are unclear as they are a relatively new product,^16^ a growing body of evidence demonstrates that they are much less harmful than smoking tobacco.^6,17^ Toxicology testing has shown that while e-cigarettes can be used to obtain similar levels of nicotine to combustible tobacco, switching to e-cigarettes can significantly reduce levels of measured carcinogens and toxins relative to smoking, with differences observed within a matter of weeks.^18–20^ Moreover, lower levels of carcinogens and toxins have been observed among long-term e-cigarette users (≥6 months) compared with current cigarette smokers.^17^

However, surveys have indicated that public perceptions of the relative harms of using e-cigarettes compared with smoking tobacco are generally inaccurate. In 2017, less than half (44%) of adults in Great Britain believed that e-cigarettes are less harmful than smoking, the lowest percentage since monitoring started in 2013.^6,7^ Just 13% correctly identified e-cigarettes as being a lot less harmful than smoking.^6,7^ The majority of adults perceived e-cigarettes to be equally harmful (23%), more harmful (3%), or were unsure about the relative risk (29%), with the percentage who believed they were more harmful the highest on record.^6,7^ Inaccurate perceptions of harm may undermine the potential public health benefits of e-cigarettes as an option for smoking cessation. If smokers are unaware that e-cigarettes offer a less harmful alternative, they may be less likely to try using e-cigarettes to quit. If dual users of e-cigarettes and cigarettes are unaware of this, they may lack the motivation to swap entirely to e-cigarettes. Thus, there is a need for effective interventions to increase the accuracy of perceptions of the relative harm associated with e-cigarettes, particularly among smokers from groups with high prevalence of smoking or low likelihood of quitting.

In addition to nationwide efforts to correct these misperceptions, Cancer Research UK, the world’s largest cancer charity, developed an advertising campaign that aimed to increase awareness of the relative harms of e-cigarettes compared with tobacco. The campaign was targeted at smokers aged 25–55 years, with a focus on those who wanted to stop and had never tried e-cigarettes. Lower socio-economic groups were also targeted because people with greater disadvantage are more likely to smoke and less likely to use e-cigarettes than those who are more advantaged^21^ (although recent data suggest this disparity is narrowing^22^). Social grade was determined using the occupation of the chief household income earner, following the National Readership Survey categorization.^23^ Disadvantage was operationalized as being C2, D, or E (C2DE) on this scale, which corresponds to manual and casual workers, state pensioners, and those who are unemployed with state benefits. Conversely, people from categories A, B, or C1 (ABC1) had clerical, managerial, administrative, and professional occupations. The campaign was piloted in Greater Manchester over a 4-week period in early 2018, with surveys conducted before and after the campaign period to evaluate changes in perceptions and use of e-cigarettes and quit attempts. For evaluation purposes, surveys were also conducted in the designated control regions Yorkshire & Humber and the North East. These comparison regions were chosen as they have a similar socio-demographic composition and smoking prevalence to Greater Manchester.

This study used data from these surveys to evaluate the extent to which the campaign was effective in:

1. Reaching members of the target group, disadvantaged (C2DE) smokers aged 25–55 years,
2. Increasing awareness of the harms of e-cigarettes relative to smoking tobacco,
3. Improving smokers’ attitudes to using e-cigarettes,
4. Promoting actual use of e-cigarettes by smokers, and
5. Increasing motivation and attempts to quit among smokers.

## Methods

### Design and Setting

Cross-sectional surveys were conducted on samples of adults (≥16 years) living in Greater Manchester (intervention region) and Yorkshire & Humber and the North East (control region) in two waves, one before (11 December 2017 to 4 January 2018) and one after (22 February 2018 to 13 March 2018) the intervention period. These were chosen as comparison regions because they share a similar demographic profile to Greater Manchester in terms of smoking prevalence, deprivation, and age and they are in a similar geographical location. All research by Cancer Research UK is carried out according to the Market Research Society Code of Conduct. This study used anonymized records and datasets available from Cancer Research UK who had already acquired appropriate permissions from participants.

### Registration

The preregistered analysis plan, data, and code used to generate results is available on the Open Science Framework (https://osf.io/uswpj/).

### Participants

Participants (*n* = 2217) were recruited via online consumer panels and incentivized (with points that were accrued across multiple surveys and could be exchanged for cash or online vouchers) to complete a roughly 15-minute online survey. Quota sampling was used to match the four survey samples (intervention and control, pre- and post-intervention) on age group (25% 16–29, 25% 30–44, 25% 45–60, 25% >60 years), sex (50% male, 50% female), social grade (based on the occupational group of the household’s chief income earner; 50% ABC1, 50% C2DE), and smoking status (19% current smoker, 25% ex-smoker, 56% nonsmoker; based on local prevalence data^24^). We also recruited a pre- and post-intervention boost sample (*n* = 580) of smokers from the targeted group (Greater Manchester residents in C2DE social grade) to facilitate a more detailed examination of the intervention’s targeting.

### Intervention

The intervention was an advertising campaign developed by Cancer Research UK that aimed to correct misperceptions about relative harms of using e-cigarettes compared with smoking tobacco. The main messages of the campaign were:

1. Research so far shows that vaping is far less harmful than smoking.
2. E-cigarettes do not contain tobacco, which is proven to cause cancer. They do contain nicotine, which is addictive, but is not responsible for the major health harms from smoking.
3. Many people are now using e-cigarettes to help them stop smoking.

The campaign’s activities included:

1. An outdoor advertising campaign, including adverts on buses, billboards, bus stops, phone kiosks and washroom posters across Greater Manchester;
2. Regional press coverage in Greater Manchester; and
3. Facebook adverts run UK-wide (excluding control regions Yorkshire & Humber and the North East).

These activities were chosen as the campaign had to adhere to Advertising Standards Authority (ASA) guidelines restricting advertising to these platforms. Street-based intercepts were used to select creative for the campaign. However, the imagery that was most popular with those surveyed was not used as it was thought to have unclear messaging about the harms of e-cigarettes relative to smoking. The campaign was piloted in Greater Manchester over a 4-week period between 15 January 2018 and 18 February 2018. Greater Manchester was selected for the pilot study as a region with particularly high smoking prevalence and deprivation levels,^25,26^ and a commitment (within the Greater Manchester Tobacco Control Plan “Making Smoking History”) to develop innovative e-cigarette friendly policies, services, and offers.^27^

### Measures

The exposure variable was region, with Greater Manchester (campaign region) coded 1 and Yorkshire & Humber and the North East (control region) coded 0.

A number of relevant outcome variables were evaluated. Recognition of the campaign materials was assessed among all respondents in Greater Manchester (including the boost sample) at follow-up by presenting them with images of the campaign poster, out of home material, social media infographics, and PR content and asking for each of the four types of content: “Before today, had you seen this content?” Those who respond yes to any of the four questions (ie, reported having seen any of the content) were coded 1 and those who respond no to all questions (ie, reported not having seen any of the content) were coded 0. Participants completed this task after answering the questionnaire so that they would not be influenced by campaign materials.

Harm perceptions of e-cigarettes were assessed among all respondents with the question: “Which of the following best describes what you think about e-cigarettes? Responses were dichotomized to distinguish between those who perceive e-cigarettes as less harmful or a lot less harmful than cigarettes and all other response options.

Perception of e-cigarettes as an effective aid to cessation was assessed among all respondents with the question: “To what extent do you think e-cigarettes are an effective aid to stop smoking regular cigarettes?”. Responses were recorded on a 6-point Likert-scale (strongly disagree to strongly agree). These were dichotomized to distinguish between those who agree that e-cigarettes are an effective aid to cessation and all other response options.

Whether respondents would consider using e-cigarettes as a cessation aid was assessed among current smokers with the question: “To what extent are you likely to consider using e-cigarettes as an aid to stop smoking regular cigarettes in the future?”. Responses were recorded on a 6-point Likert-scale (very unlikely to very likely). These were dichotomized to distinguish between those who would be likely to use e-cigarettes as an aid to cessation and all other response options.

Whether respondents would recommend e-cigarettes as a cessation aid to someone else was assessed among all respondents with the question: “To what extent are you likely to recommend e-cigarettes to someone you know as an aid to stop smoking regular cigarettes?” Responses were recorded on a 6-point Likert-scale ranging (very unlikely to very likely). These were dichotomized to distinguish between those who would be likely to recommend e-cigarettes as an aid to cessation and all other response options.

E-cigarette use was assessed among current and ex-smokers with the question: “Have you tried an electronic cigarette or vaping device in the last two months?” with yes coded 1 and no coded 0. Frequency of current e-cigarette use was assessed among those who report having used an e-cigarette in the last 2 months with the question: “How often, if at all, do you currently use an electronic cigarette or vaping device?” Responses were dichotomized to distinguish between those who use e-cigarettes daily and all other response options.

Motivation to stop smoking was assessed among current smokers using the Motivation to Stop Scale,^28^ Responses were dichotomized to distinguish between those with high motivation (ie, intend to stop within the next 3 months) and all other response options.^29,30^

Quit attempts were assessed among current and ex-smokers with the question: “Have you been attempting to quit smoking in the last 4 to 8 weeks? Please include any attempts you’re currently making” with yes coded 1 and no coded 0.

Covariates included: age group (16–29, 30–44, 45–60, >60 years), sex (male, female), and social grade (ABC1, C2DE). Smoking status was measured by asking “Which of the following BEST applies to you?”. Those who responded “I smoke, but not everyday” or “I smoke everyday” were considered current smokers and all others were considered nonsmokers.

### Analyses

We used descriptive statistics to summarize responses to the outcome variables in the intervention and control regions before and after the intervention. Differences in recognition of the campaign materials at follow-up between target (ie, age 25–55 years and social grades C2DE) and nontarget groups (ie, age <25 or >55 and social grades ABC1) were analyzed using logistic regression. This analysis included participants from the boost sample. Interactions between time (pre-intervention, post-intervention) and region (campaign, control) for all outcomes of interest were analyzed using generalized estimating equations. Participants recruited as part of the boost sample were excluded to maintain comparability of the intervention and control samples. Among all respondents, we tested whether there was a greater change in the intervention region versus the control region from pre- to post-intervention in perceptions of e-cigarettes. We also tested the change in perceptions of e-cigarettes among a sample of current smokers, alongside other relevant outcomes such as motivation to quit, e-cigarette use, and quit attempts. Among current smokers who reported using e-cigarettes at all in the past 2 months, we tested whether there was a greater change in daily e-cigarette use. All analyses controlled for age, sex, and social grade.

We calculated Bayes factors (BF) for nonsignificant interactions to determine whether they provide evidence for no effect (BF < 1/3) when compared to the alternative hypothesis or indicate data insensitivity (BF ≥ 1/3 and < 3). The alternative hypothesis was modeled as a half-normal distribution centered on zero, with a standard deviation equal to the expected effect size. The expected effect size (odds ratio [OR] = 1.24) was used based on previous literature.^31,32^ Sensitivity analyses were performed using smaller (OR = 1.10) and larger (OR = 1.40) expected effect sizes to test how robust conclusions were to changes in the alternative hypothesis.

## Results

Socio-demographic characteristics of the samples in each region pre- and post-campaign are shown in Table 1. Over a third (36.7%, 95% CI 33.0%–40.6%) of participants in the post-campaign Manchester sample recognized at least one of the campaign materials, compared with a fifth (19.6%, CI 16.0%–23.7%) in the control region. There was no significant difference in recognition between target participants (C2DE smokers aged 25–55 years) and others (38.3% vs. 35.8%, OR = 1.17, 95% CI 0.83–1.65). The aspect of the campaign with the largest reach was the bus stop poster with 28.7% of participants reporting recognizing it.

**Table 1.** Sample Characteristics

	Control regions		Campaign region	
	Before, % (*n*)	After, % (*n*)	Before, % (*n*)	After, % (*n*)
Smoking status				
Nonsmoker	73.6 (309)	80.4 (325)	63.0 (260)	81.3 (325)
Smoker	26.4 (111)	19.6 (79)	37.0 (153)	18.8 (75)
Social Grade				
ABC1	43.6 (183)	50.2 (203)	42.6 (176)	50.8 (203)
C2DE	56.4 (237)	49.8 (201)	57.4 (237)	49.3 (197)
Age (years)				
16–29	18.3 (77)	23.5 (95)	21.1 (87)	24.5 (98)
30–44	28.8 (121)	26.5 (107)	31.5 (130)	26.3 (105)
45–60	35.2 (148)	24.5 (99)	33.4 (138)	25.3 (101)
>60	17.6 (74)	25.5 (103)	14.0 (58)	24.0 (96)

Table 2 shows changes in outcomes over time by region. In the general population, interactions between time and region were nonsignificant for all outcomes except for perception of e-cigarettes as effective cessation aids, with smaller increases from pre- to post-campaign in the campaign region (49.9% to 54.0%) compared with the control region (40.5% to 55.0%; OR = 0.66, 95% CI .45 – .98, *p* = .04). In current smokers, the only significant time by region interaction was for motivation to quit, which increased in the intervention region (44.0% to 48.0%) but decreased in the control region (40.5% to 21.5%; OR = 2.97, 95% CI 1.25–7.16, *p* = .01).

**Table 2.** Results of Generalized Estimating Equations Testing Interactions Between Region (Campaign, Control) and Time (Pre- and Post-Campaign) for E-Cigarette Perceptions and Use, Motivation to Quit, and Incidence of Quit Attempts

	Control region, % (*n*)	Campaign region, % (*n*)	Main effect region, OR (95% CI)	Main effect time, OR (95% CI)	Interaction group and time, OR (95% CI)
Outcomes among all participants					
Perceptions of e-cigarettes as less harmful than conventional cigarettes					
Pre	48.3 (203)	55.0 (227)			
Post	57.5 (234)	57.8 (231)	1.29 (0.98–1.69), *p* = .07	1.46 (1.10–1.92), *p* < .01	0.76 (0.51–1.13), *p* = .18, BF = 0.36
Perception of e-cigarettes as effective cessation aid					
Pre	40.5 (170)	49.9 (206)			
Post	55.0 (222)	54.0 (216)	1.44 (1.10–1.90), *p* < .01	1.82 (1.38–2.41), *p* < .001	0.66 (0.45–0.98), *p* = .04
Likely to recommend e-cigarettes as cessation aid					
Pre	28.6 (120)	34.4 (142)			
Post	29.2 (118)	36.0 (144)	1.26 (0.94–1.70), *p* = .13	1.04 (0.76–1.41), *p* = .82	1.07 (0.70–1.64), *p* = .74, BF = 0.86
Outcomes among smokers					
Perceptions of e-cigarettes as less harmful than conventional cigarettes					
Pre	43.2 (48)	32.9 (26)			
Post	56.8 (63)	67.0 (53)	0.91 (0.55–1.50), *p* = .71	3.00 (1.24–7.60), *p* = .02	0.97 (0.41–2.3), *p* = .95, BF = 0.88
Perception of e-cigarettes as effective cessation aid					
Pre	51.4 (57)	54.2 (83)			
Post	55.7 (44)	64.0 (48)	1.08 (0.65–1.78), *p* = .76	0.65 (0.23–1.79), *p* = .40	1.36 (0.59–3.17), *p* = .47, BF = 1.18
Likely to use e-cigarette as cessation aid					
Pre	42.3 (47)	49.7 (76)			
Post	49.4 (39)	56.0 (42)	1.32 (0.80–2.2), *p* = .28	0.98 (0.35–2.72), *p* = .97	0.96 (0.42–2.20), *p* = .92, BF = 0.86
Likely to recommend e-cigarettes as cessation aid					
Pre	38.7 (43)	43.8 (67)			
Post	40.5 (32)	56.0 (42)	1.2 (0.72–2.01), *p* = .47	1.09 (0.59–2.02), *p* = .78	1.44 (0.63–3.32), *p* = .39, BF = 1.25
High motivation to stop smoking					
Pre	40.5 (45)	44.0 (67)			
Post	21.5 (17)	48.0 (36)	1.11 (0.67–1.84), *p* = .68	0.38 (0.19–.74), *p* < .01	2.97 (1.25–7.16), *p* = .01
Used E-cigarette in past 2 months					
Pre	41.5 (44)	44.1 (63)			
Post	42.5 (31)	56.7 (38)	1.12 (0.67–1.88), *p* = .67	1.10 (0.59–2.06), *p* = .76	1.51 (0.64–3.60), *p* = .35, BF = 1.29
Make a quit attempt in past 2 months					
Pre	29.7 (33)	41.8 (64)			
Post	30.4 (24)	54.7 (41)	1.67 (0.99–2.85), *p* = .06	0.97 (0.50–1.85), *p* = .93	1.60 (0.68–3.79), *p* = .28, BF = 1.38
Use e-cigarettes daily					
Pre	13.0 (6)	28.9 (22)			
Post	28.2 (11)	35.7 (15)	2.37 (0.97–6.20), *p* = .07	1.55 (0.50–4.79), *p* = .44	0.78 (0.19–3.17), *p* = .72, BF = 0.88

OR = odds ratio; CI = confidence interval; BF = Bayes factor.

Bayes factors were calculated for nonsignificant results to determine whether the data were insensitive or provided evidence for no effect. All Bayes factors were between a third and three, suggesting that there was insufficient evidence to determine the impact of the campaign across these outcomes. Sensitivity analyses that used both higher and lower expected effect sizes provided the same conclusion for all but one of the interactions (Supplementary Material), suggesting that there was insufficient evidence to reject or accept either the null or alternative hypotheses.

## Discussion

An awareness campaign to improve harm perceptions of e-cigarettes relative to smoking, developed by Cancer Research UK, was recognized by over a third of participants in a post-campaign evaluation. Recognition was not significantly higher in the target group of low socio-economic status (C2DE) smokers aged 25–55 years. When compared with smokers from a control region, smokers who were exposed to the campaign reported a more positive change in motivation to quit from before to after the campaign was run. However, among all participants, perceptions of e-cigarettes as an effective cessation aid improved more in the control than campaign region. There was insufficient evidence to determine the impact of the campaign on other outcomes, including the campaign’s primary objective: harm perceptions of e-cigarettes relative to smoking.

Past mass media campaigns have successfully altered the public’s attitudes towards smoking, improved motivation to quit smoking, and reduced smoking prevalence.^33^ This campaign also aimed to promote quitting through the most common cessation aid in the United Kingdom: e-cigarettes.^8^ Given that the campaign only ran for 1 month, reach was impressive with more than one in three of those surveyed recognizing at least one of the advertisements. This is comparable to the level of recognition recorded from other smoking cessation campaigns than ran for a much longer duration.^34,35^ The similarly high level of recognition found among C2DE smokers aged 25–55 years and other populations suggest that, while the campaign was effective at reaching this target group, it was similarly successful at reaching other groups. The relatively high level of recognition of campaign materials in the control region could have resulted from two issues. Firstly, some of the participants in the control region may have been exposed to these materials, either by traveling to Greater Manchester or through social media. If this happened, the effect of the campaign on outcomes would be underestimated. Second, participants may have mistaken these materials for other similar messaging that they have been exposed to. For instance, over the same time period, Public Health England ran a nationwide campaign on e-cigarettes. If this occurred, the same is likely to have happened in the campaign region and, thus, the true number of people who recognized these materials would have been overestimated.

Motivation to quit smoking tends to be high at the start of a new year and subsequently drops off throughout January.^36^ We found that those in the campaign region were insulated from this drop. The campaign’s promotion of e-cigarettes as a cessation aid, which are widely available and often quicker to access than other cessation aids such as pharmacotherapy on prescription, may have contributed to this. Motivation to quit is a strong predictor of future quit attempts.^37^ Therefore, if the difference in motivation between regions was caused by the campaign, there is a rationale to expand it to other regions. However, other unmeasured influences may also have contributed towards these different trends across regions.

Perceptions of e-cigarettes as effective cessation aids increased in both regions from pre- to post-campaign, but showed a significantly greater increase in the control region. At baseline, perceptions of e-cigarettes as effective cessation aids were much higher in the campaign region. Therefore, the smaller increase in this region may represent a ceiling effect, where the high initial level of this variable meant that it was unresponsive to interventions aimed to raise it. Conversely, low baseline effectiveness perceptions in the control region meant other campaigns aimed at correcting these misperceptions—such as one run by Public Health England^38^ over the same time period—would likely have had a greater impact, resulting in similar levels of perceptions at follow-up.

Bayes factors indicated data were insensitive to detect significant associations for most outcomes. Effects of mass media advertising campaigns tend to be small, especially if they are run for a short period of time.^33^ Therefore, the lack of evidence to determine whether changes in the other outcomes differed between regions was likely due to a lack of statistical power. We may have been able to detect the campaign’s impact on these outcomes if it ran for a longer time period, or if the sample size was larger.

A strength of this study was that we were able to adjust for trends in outcomes over time by using a comparison region containing populations who were not exposed to the campaign. A pre-post analysis solely in the campaign region would have been unable to determine what effects were caused by seasonal changes, which are especially variable from December to February.^36^ Nonetheless, the study design used had several limitations. Most significantly, we had to assume that the trends in outcomes would be identical across groups, were it not for the influence of the advertising campaign. For this to be true, any unmeasured variables that influence outcomes must either be constant over time or equal across groups, which is unlikely to be the case. For instance, over the campaign period, there were other interventions in place (in addition to the Cancer Research UK campaign) that aimed to alter public perceptions of e-cigarettes.^38^ These may have had a greater impact on outcomes in the control region due to lower baseline perceptions. Moreover, there is regular news coverage both supporting and opposing e-cigarette use, which could also have differentially affected trends across regions. To better account for these unobserved variables, a longer time-series analysis or cluster randomized-controlled trial would be required. Another limitation is that all data were self-reported, which introduces scope for error and bias. However, due to the low demand characteristics in the study, this is unlikely to significantly influence our results. Recall of the campaign materials was assessed with cued recall, which may have biased estimates of recognition upwards. Issues also arise from the sample used. Firstly, unlike a true pre-post study, different individuals were recruited in baseline and follow-up samples. This introduces bias as these samples may vary on important characteristics. Secondly, sample size was insufficient to detect even relatively large changes in outcomes. Future research, with a larger sample and more robust design, would be useful to determine whether similar advertising campaigns can impact harm perceptions towards e-cigarettes when compared to smoking.

## Conclusions

A regional educational advertising campaign in England was associated with increased motivation to quit among smokers exposed to the campaign, when compared with those in an unexposed region. It was also associated with a smaller increase in perceptions of e-cigarettes as an effective cessation aid among the general population, which may be due to different baseline perceptions across regions. Bayes factors showed that the impact of the campaign on the other outcomes was inconclusive, which may indicate that the campaign needed to run across a longer period to cause a detectable effect.

## Funding

The educational advertising campaign and data collection were funded by Cancer Research UK (CRUK). SEJ’s salary is supported by CRUK (C1417/A22962) and the Economic and Social Research Council (ES/R005990/1). CI is employed by CRUK. LB is seconded part-time to CRUK in an advisory role and her employer (the University of Edinburgh) receives funding from CRUK for a portion of her time. HT-B’s salary is funded by an unrestricted grant provided by Global Research Award in Nicotine Dependence (GRAND) under the GRAND scheme.

## Declaration of Interests

LS has received honoraria for talks, an unrestricted research grant and travel expenses to attend meetings and workshops from Pfizer, and has acted as paid reviewer for grant awarding bodies and as a paid consultant for health care companies. All authors declare no financial links with tobacco companies or e-cigarette manufacturers or their representatives.

## Supplementary Material

ntz236_suppl_Supplementary-Material
