# Supplementary material for: Evaluation of the Impact of a Regional Educational Advertising Campaign on Harm Perceptions of E-Cigarettes, Prevalence of E-Cigarette Use, and Quit Attempts Among Smokers
Source: Nicotine Tob Res. 2019 Dec 14;22(7):1148–54. doi: 10.1093/ntr/ntz236 (PMC7291809; doi:10.1093/ntr/ntz236)
Supplement: ntz236_suppl_Supplementary-Material [file ntz236_suppl_supplementary-material.pdf]

Supplementary Material

Table S1: Bayes factors for non-significant results, using different expected effect sizes when modelling the alternative hypothesis.

	Bayes Factor (OR = 1.10)	Bayes Factor (OR = 1.24)	Bayes Factor (OR = 1.40)
**Outcomes among all participants**			
Perceptions of e-cigarettes as less harmful than conventional cigarettes	0.62	0.37	0.26*
Likely to recommend e-cigarettes as cessation aid	1.02	0.86	0.69
**Outcomes among smokers**			
Perceptions of e-cigarettes as less harmful than conventional cigarettes	0.98	0.88	0.77
Perception of e-cigarettes as effective cessation aid	1.11	1.18	1.17
Likely to use e-cigarette as cessation aid	0.96	0.86	0.75
Likely to recommend e-cigarettes as cessation aid	1.14	1.25	1.27
Used E-cigarette in past 2 months	1.15	1.29	1.34
Make a quit attempt in past 2 months	1.19	1.38	1.47
Use e-cigarettes daily	0.96	0.88	0.81

Notes: *OR* odds ratio, * moderate evidence for null hypothesis.
